# The Influence of High and Low Doses of Bisphenol A (BPA) on the Enteric Nervous System of the Porcine Ileum

**DOI:** 10.3390/ijms19030917

**Published:** 2018-03-20

**Authors:** Kamila Szymanska, Krystyna Makowska, Slawomir Gonkowski

**Affiliations:** Department of Clinical Physiology, Faculty of Veterinary Medicine, University of Warmia and Mazury, Oczapowskiego Str. 13, 10-719 Olsztyn, Poland; krystyna.makowska@uwm.edu.pl (K.M.); slawomir.gonkowski@uwm.edu.pl (S.G.)

**Keywords:** phenols, digestive tract, ileum, enteric nervous system, pig

## Abstract

Bisphenol A, used in the production of plastic, is able to leach from containers into food and cause multidirectional adverse effects in living organisms, including neurodegeneration and metabolic disorders. Knowledge of the impact of BPA on enteric neurons is practically non-existent. The destination of this study was to investigate the influence of BPA at a specific dose (0.05 mg/kg body weight/day) and at a dose ten times higher (0.5 mg/kg body weight/day), given for 28 days, on the porcine ileum. The influence of BPA on enteric neuron immunoreactive to selected neuronal active substances, including substance P (SP), vasoactive intestinal polypeptide (VIP), galanin (GAL), vesicular acetylcholine transporter (VAChT—used here as a marker of cholinergic neurons), and cocaine- and amphetamine-regulated transcript peptide (CART), was studied by the double immunofluorescence method. Both doses of BPA affected the neurochemical characterization of the enteric neurons. The observed changes depended on the type of enteric plexus but were generally characterized by an increase in the number of cells immunoreactive to the particular substances. More visible fluctuations were observed after treatment with higher doses of BPA. The results confirm that even low doses of BPA may influence the neurochemical characterization of the enteric neurons and are not neutral for living organisms.

## 1. Introduction

Bisphenol A (BPA) is an organic compound that is widely used in the manufacture of plastic, among others in the production of bottles, toys, food containers, and medical dental products [1,2]. Since BPA is able to leach into food from polycarbonate containers, humans are exposed to this substance mainly through the digestive tract [2]. BPA is absorbed by the gastrointestinal (GI) tract and distributed around the body via the bloodstream. To date, BPA has been detected in multiple tissues and liquids of human and animal organisms, including urine, saliva, breast milk, adipose tissue, reproductive tissues, and fetuses [3,4,5].

Previous studies have shown that bisphenol A may have various negative effects on living organisms due mainly to the structural similarities of this substance to estrogen [6,7]. BPA displays high-affinity binding to estrogen receptors and, above all, can have negative effects on the reproductive system [8] and metabolism [9]. It is known that even a short exposure to BPA during the prenatal and early periods of life can induce changes in the growth of reproductive organs and may change hormone levels, which may last a lifetime [10].

BPA may also affect the nervous system. It changes nociception and pain stimuli conduction [11], inhibits estradiol-induced hippocampal synaptogenesis [6], and may affect the calcium concentration within neurons [12]. Moreover, some investigations have described the neurotoxic properties of BPA, characterized by disorders in behavior, learning, and memory in rodents [13,14,15]. Besides, it has been described that BPA can result in the remodeling of synaptic endings and disorders of higher cognitive functions [16]. It is known that even a single exposure to bisphenol A in the periparturient period can change the levels of proteins important for brain physiology (such as Ca2+/calmodulin-dependent protein kinases II (CaMKII) and synaptophysin) in adult rodents [17]. In turn, in vitro studies have described BPA as a neurotoxic factor, which affects the development of dendrites and synapses of hypothalamic cells and inhibits neurite extension [18,19].

Another system influenced by BPA is the immune system [20]. It is known that BPA can inhibit lymphocyte mitogenesis [21] and macrophage adhesion [22]. BPA also affects the action of CD4+ lymphocytes (causing an increase in IFN γ, IL-4, and IL-10 production), B-lymphocytes (resulting in an increase in IgG2a and IgA secretion), and macrophages (decreasing TNF-α secretion) [23].

Knowledge concerning the impact of BPA on the stomach and intestine is rather limited. It is known that BPA may alter the intestinal barrier function, gut nociception, and expression of genes [24]. Moreover, BPA may be involved in processes associated with gastrointestinal inflammation [25,26]. Some studies have also suggested that perinatal or early (in juvenile period) exposure to xenoestrogens (including BPA) is a factor predisposing to various gastrointestinal diseases in later life [7]. However, several aspects connected with the effects of BPA (especially in low doses) on the digestive tract remain incompletely elucidated.

**Figure 1:**
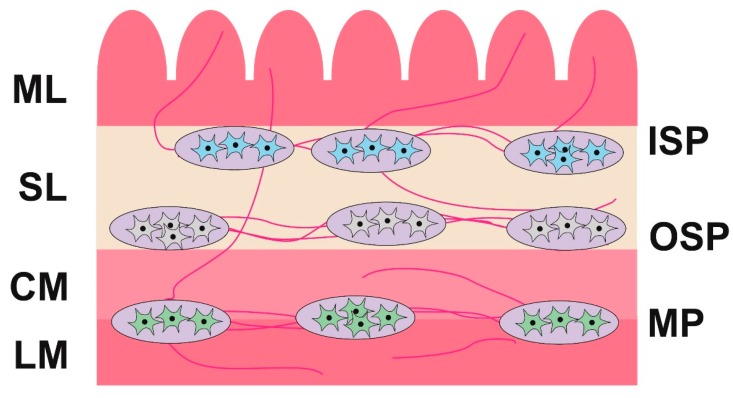
The enteric nervous system in the ileum of the pig. Parts of the wall of the intestine: LM—longitudinal muscle layer; CM—circular muscle layer; SL—submucosal layer; ML—mucosal layer. Parts of the enteric nervous system: MP—myenteric plexus; OSP—outer submucous plexus; ISP—inner submucous plexus.

One of these is the influence of BPA on the enteric nervous system (ENS). It is known that the ENS is located in the intestinal wall and in large mammal species (including the pig) it consists of three types of intramural plexuses: the myenteric plexus (MP) (between the longitudinal and circular muscle layers), the outer submucous plexus (OSP) (on the inner side of the circular muscle layer), and the inner submucous plexus (ISP) (near the lamina propria of the mucosal layer) (Figure 1) [27]. The ENS shows a significant independence from the central nervous system and regulates the majority of functions of the digestive tract, under both physiological conditions and during pathological processes. Besides, the ENS is one of the first barriers of the organism against toxins in food. It is involved in homeostasis maintenance and mechanisms connected with pathological changes in the intestine. In turn, fluctuations in the neurochemical characteristics of the intestinal neuronal cells can be the first subclinical symptoms of various pathological processes [28].

The aim of this investigation was to study the impact of low and high doses of BPA on the ENS of the porcine ileum. The first reports on the influence of bisphenol A on the hormonal balance date back to the 1930s and the first assessment of exposure to bisphenol A was made in 1986 [29]. Since then, as research has progressed, new legal regulations regarding BPA have been introduced. Some countries, such as Canada, Japan, and South Korea, have banned the use of bisphenol A [29,30]. Other countries, including countries belonging to the European Union, have banned the use of BPA in products that come into contact with food intended for children (such as bottles or teats) [29]. In the European Union, a ban on the use of BPA for the production of baby bottles, as well as the import of such products, has been in force since 2011. It should be noted, however, that many aspects related to the impact of BPA on living organisms are not fully elucidated, and work on determining the effects of long-term exposure to bisphenol A is ongoing [10]. Nevertheless, previous studies have proven that BPA has a multidirectional negative impact on the health of both humans and animals [2,6].

The selection of an experimental animal species was not accidental. The domestic pig, because of its neurochemical, physiological, and immunological resemblance to the human, is considered an adequate animal model for studies on cardiovascular diseases, metabolic disorders, and injury and repair processes [31,32,33]. Moreover, humans and pigs have similar enteric nervous systems, intestinal functions, and adaptive processes in response to pathological factors [28,34]. The results of this investigation may be treated as an animal model for the impact of BPA on the human GI tract.

## 2. Results

Experimental animals after BPA administration did not show any clinical symptoms of intoxication. Behavior, appetite, and increments in body weight of animals treated with BPA were similar to those in the control animals.

### 2.1. Morphological Changes

Macroscopic examination performed immediately after euthanasia showed no differences between the control group (C group) and the low dose group (LD group), whilst hyperemia and petechiae were observed in the mucosa from the high dose group (HD group).

**Figure 2:**
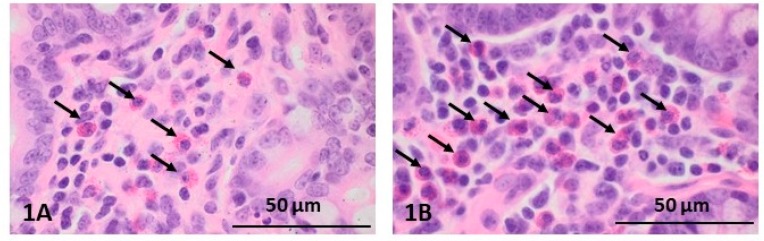

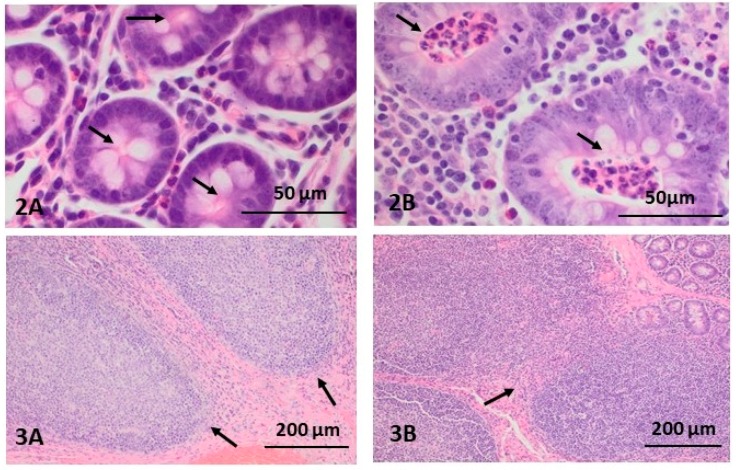
Histopathological staging of the ileum after administration of low (A) and high (B) doses of BPA: (1) less (**1A**) and more numerous (**1B**) eosinophils (indicated by arrows); (2) the lack (**2A**) and the presence (**2B**) of inflammatory cells in intestinal crypts (intestinal crypts are indicated by arrows); (3) the changes in the appearance of Peyer’s patches depending on BPA dose: Peyer’s patches in the LD group (**3A**) and merged structures in the HD group (**3B**) (Peyer’s patches are indicated by arrows).

Histopathological examination of intestinal sections from all groups showed that their structure was preserved and the villi were of the proper structure. Plasmatic cells and eosinophilic granulocytes were present in the mucosal and submucosal lining. However, the number of eosinophils in animals of the HD group (up to 100 eosinophils in the large field of view, 40×) was higher than in pigs of the control and LD groups (Figure 2(1A, 1B)). Furthermore, only in the animals receiving the high dose were inflammatory cells in the lumen of intestinal crypts visible (Figure 2(2B)); in the other groups of animals, these cells were not observed (Figure 2(2A)).

Histopathological dissimilarities between the groups of animals also consisted of a difference in the size of the ileal Peyer’s patches, which in the HD group additionally formed merged structures (Figure 2(3A,3B)).

### 2.2. Changes in Neurochemistry in the ENS

**Figure 3:**
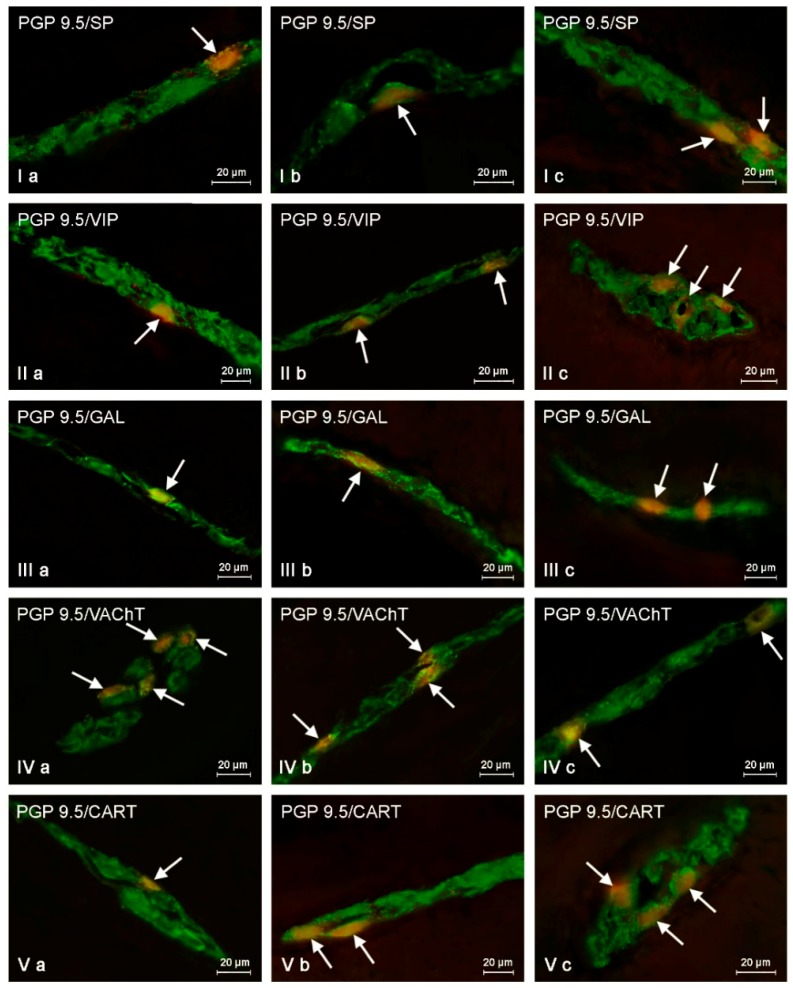
Myenteric plexus in control animals (**a**) and after BPA treatment in low (**b**) and high (**c**) doses, immunostained for protein gene product 9.5 (PGP 9.5, a pan-neuronal marker) and other neuronal factors including substance P (SP), vasoactive intestinal polypeptide (VIP), galanin (GAL), vesicular acetylcholine transporter (VAChT—used here as a marker of cholinergic neurons), and cocaine- and amphetamine-regulated transcript peptide (CART). Neurons showing co-localization of PGP 9.5 and other neuronal factors are indicated with arrows.

**Figure 4:**
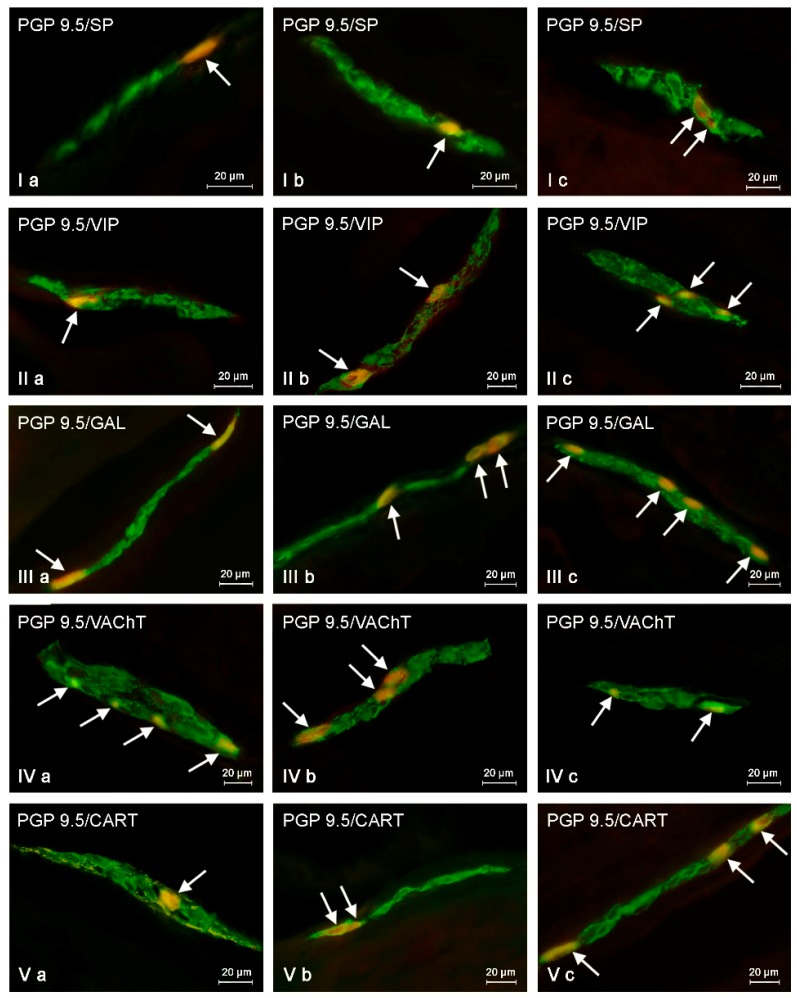
Outer submucous plexus in control animals (**a**) and after BPA treatment in low (**b**) and high (**c**) doses, immunostained for protein gene product 9.5 (PGP 9.5, a pan-neuronal marker) and other neuronal factors including substance P (SP), vasoactive intestinal polypeptide (VIP), galanin (GAL), vesicular acetylcholine transporter (VAChT—used here as a marker of cholinergic neurons), and cocaine- and amphetamine-regulated transcript peptide (CART). Neurons showing co-localization of PGP 9.5 and other neuronal factors are indicated with arrows.

**Figure 5:**
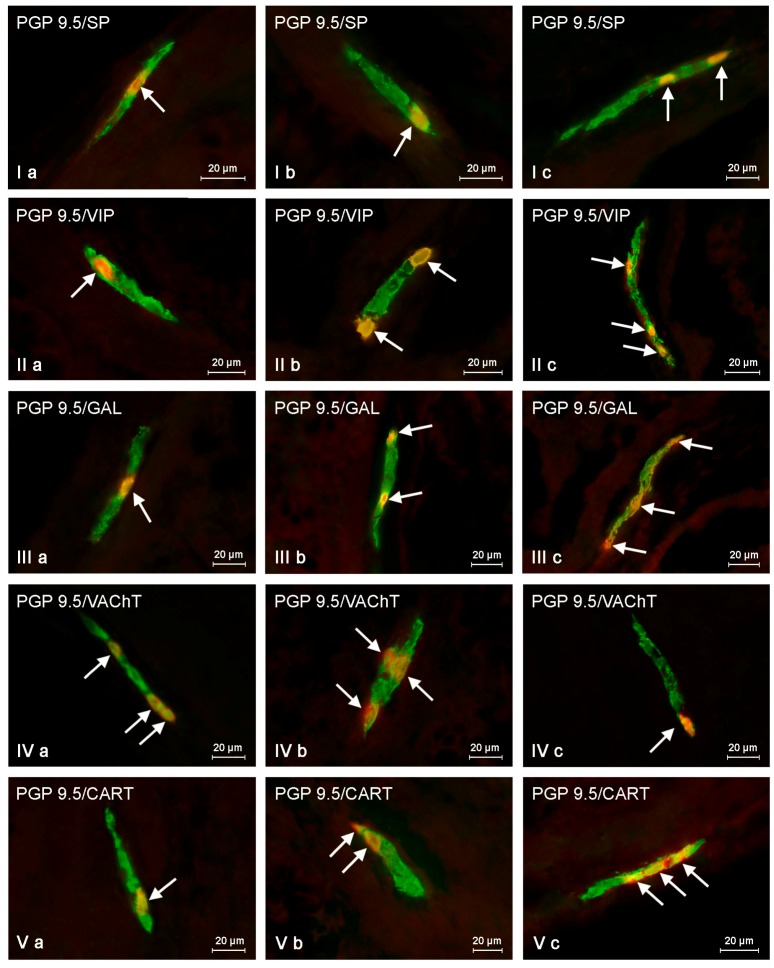
Inner submucous plexus in control animals (**a**) and after BPA treatment in low (**b**) and high (**c**) doses, immunostained for protein gene product 9.5 (PGP 9.5—used here as a pan-neuronal marker) and other neuronal factors including substance P (SP), vasoactive intestinal polypeptide (VIP), galanin (GAL), vesicular acetylcholine transporter (VAChT—used here as a marker of cholinergic neurons), and cocaine- and amphetamine-regulated transcript peptide (CART). Neurons showing co-localization of PGP 9.5 and other neuronal factors are indicated with arrows.

Both low and high doses of toxin resulted in fluctuations in the percentage of enteric neurons immunoreactive to particular substances, and the character and severity of these fluctuations varied depending on the type of enteric plexus and active substance studied, as well as the dose of BPA (Table 1, Figure 3, Figure 4 and Figure 5).

#### 2.2.1. Myenteric Plexus (MP)

Under physiological conditions in the MP (Figure 3), the presence of VAChT was noted in 18.35 ± 0.21% of all neuronal cells immunoreactive to PGP 9.5. A slightly lower percentage of neurons exhibited immunoreactivity to VIP and/or SP (15.05 ± 0.17% and 11.01 ± 0.16%, respectively). However, the percentage of neuronal cells immunoreactive to GAL and/or CART did not exceed 10% (7.91 ± 0.04% and 7.58 ± 0.39%, respectively) (Table 1).

Low doses of BPA caused a slight increase in the number of CART-like immunoreactive (LI) neuronal cells (to 10.70 ± 0.40%) and an insignificant decrease in the percentage of neurons showing the presence of VAChT (to 17.54 ± 0.24%) (Figure 3). For the other neuronal active factors investigated, the disparities between control and LD groups were not statistically significant (Table 1).

High doses of BPA caused fluctuations in the percentage of myenteric neurons immunopositive to all substances investigated (Table 1, Figure 3). The most obvious fluctuations were observed in the case of neurons immunoreactive to CART (the increase to 17.09 ± 0.52%, of about 9.5 percentage points (pp)). Less distinct changes were noted in cells showing the presence of GAL (the increase to 13.40 ± 0.39%, about 5 pp) and/or SP (the increase to 15.15 ± 0.28%, about 4 pp). High doses of BPA also caused a slight increase in the number of neurons showing the presence of VIP (to 17.51 ± 0.04%, about 2.5 pp). In turn, the percentage of VAChT-LI was lower than in control animals and achieved 13.67 ± 0.18% (a decrease of about 4.6 pp).

#### 2.2.2. Outer Submucous Plexus (OSP)

Under physiological conditions, VAChT-LI neurons accounted for 19.93 ± 0.10% of all OSP neuronal cells. The percentage of perikarya showing the presence of VIP, SP, and/or GAL amounted for 10.57 ± 0.39%, 9.76 ± 0.12%, and 9.60 ± 0.41%, respectively. The smallest population of enteric neuronal cells was immunoreactive to CART (4.47 ± 0.11%) (Table 1; Figure 4).

Low doses of BPA resulted in an increase in the percentage of neurons immunoreactive to GAL (to 12.92 ± 0.42%, about 3 pp) and/or VIP (to 12.61 ± 0.11%, about 2 pp), while the number of VAChT -LI neurons was lower than in control animals (a decrease to 18.24 ± 0.23, approx. 1.6 pp) (Figure 4). The percentage of neuronal cells showing the presence of SP and CART amounted to 10.39 ± 0.32% and 5.29 ± 0.36%, respectively, and the differences in these values between the LD group and control animals were not statistically significant (Table 1).

In the HD group, the most obvious increase was noted for perikarya immunoreactive to GAL (to 18.08 ± 0.35%, about 8.5 pp) and/or SP (to 17.73 ± 0.33%, about 8 pp). A slightly less pronounced increase was noted in the percentage of VIP- and/or CART-LI neurons, which amounted to 16.00 ± 0.08% and 9.65 ± 0.20%, respectively (Figure 4). In both cases, the fluctuations reached about 5 pp. The percentage of VAChT in the HD group was lower than that observed in control animals and amounted to 14.70 ± 0.12% (a decrease of about 5 pp) (Table 1).

#### 2.2.3. Inner Submucous Plexus (ISP)

In control animals, VAChT was noted in 26.42 ± 0.38% of all PGP9.5-LI neurons. GAL-like immunoreactivity was noted in fewer perikarya (14.14 ± 0.13%). The percentage of neurons showing the presence of SP and/or VIP was even smaller and amounted to 9.32 ± 0.37% and 7.64 ± 0.17%, respectively. In turn, the immunoreactivity to CART was observed in only 3.80 ± 0.16% of neurons (Table 1; Figure 5).

Low doses of BPA caused an increase in the percentage of neurons immunoreactive to GAL (to 18.17± 0.08%, about 4 pp), VIP (10.85 ± 0.34%, about 3 pp), and/or CART (5.30 ± 0.24%, 1.5 pp). Moreover, low doses of BPA caused a decrease in the percentage of VAChT-LI neurons (to 25.21 ± 0.23%, about 1.2 pp). In turn, the number of SP-LI neurons (10.32 ± 0.31%) was not statistically distinct from the values noted in pigs of C group (Figure 5).

In the HD group, the clearest increase was observed in neurons immunoreactive to VIP (to 17.80 ± 0.28%, about 10 pp) and/or GAL (22.72 ± 0.30%, about 8.5 pp) (Figure 5). Less visible fluctuations were observed for SP-LI neurons (increase to 15.83 ± 0.42%, about 6.5 pp) and cells immunoreactive to CART (increase to 8.02 ± 0.04%, about 4 pp). In turn, the percentage of VAChT-LI neurons achieved 20.70 ± 0.11% and was approx. 5.7 pp lower than in pigs under physiological conditions (C group) (Table 1).

### 2.3. Changes in Cytokine Levels

**Figure 6:**
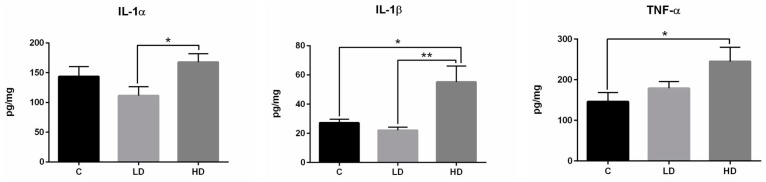
The concentration of the selected pro-inflammatory cytokines in the ileal Payer’s patches in control animals (C) and under low (LD) and high (HD) doses of BPA. Statistically significant (*p* ≤ 0.05) and highly statistically significant (*p* ≤ 0.01) differences between C group and LD group, C group and HD group, as well as LD group and HD group, are marked with * and **, respectively.

During the present study, the main pro-inflammatory cytokine levels (including TNF-α, IL-1α, and IL-1β) within the ileal Peyer’s patches were determined to designate the actual immune response profile (Figure 6). In the C group, the concentration of TNF-α, IL-1α, and IL-1β amounted to 146.24 ± 22.01 pg/mg, 143.73 ± 16.75 pg/mg, and 27.18 ± 2.52 pg/mg, respectively. Low doses of BPA did not cause statistically significant changes in the levels of the cytokines studied with regard to group C. The values amounted to 178.79 ± 16.52 pg/mg (TNF-α), 111.65 ± 14.72 pg/mg (IL-1α), and 22.13 ± 2.13 pg/mg (IL-1β). High doses of BPA resulted in an increase in the concentration of the cytokines studied in comparison to the C group. These values amounted to 245.11 ± 34.96 pg/mg, 167.70 ± 14.06 pg/mg and 55.14 ± 10.90 pg/mg for TNF-α, IL-1α, and IL-1β, respectively.

## 3. Discussion

This study confirms, as known from previous studies [27,28,35], the complex construction of the porcine ENS and the diversity of enteric neurons in terms of neurochemical characterization. Until now, besides acetylcholine—the main neuromediator of the ENS—a broad spectrum of other active neuronal factors have been described in intestinal neurons [36,37,38]. These substances most frequently are neuromediators and/or neuromodulators and take part in the regulation of various intestinal functions, such as gastrointestinal motility, secretory activity of the gastrointestinal tract, intestinal blood flow, absorption of nutrients, and many other functions. Moreover, during this study, some similarities between porcine intestinal innervation and the previously described neurochemical organization of the human ENS [39,40] were noted, which is in agreement with earlier observations [38] and strongly indicates that the domestic pig can be an animal model for the human ENS. In turn, changes in the neurochemical character observed under the influence of BPA confirm the adaptability of the ENS to changes during pathological processes and toxin exposure [36,38].

The results also confirmed the adaptability of the ENS to changes connected with the impact of toxic substances. Previous studies have described how enteric neurons may change their neurochemical characterization, morphology, functions, and electrophysiological properties under the influence of physiological factors (such as an ontogenesis or changes in diet) and numerous pathological processes taking place inside and outside of the GI tract [28,40,41]. Most frequently, the above-mentioned changes are the result of processes connected with adaptive and/or neuroprotective reactions, which are intended to maintain homeostasis in the intestine under conditions altered by the acting stimulus.

Differences in the distribution of the substances studied between the particular types of enteric plexuses observed under both physiological conditions and after BPA administration are probably connected with various functions of the enteric neurons, depending on their localization. Previous studies have shown that the myenteric plexus is primarily responsible for the regulation of intestinal motility [35,42], and the neurons located in the inner submucous plexus are primarily involved in processes connected with mucosal layer activity, including the secretion of enzymes and hormones and/or the absorption of nutrients [35,43]. In turn, cells of the outer submucous plexus take part in the control of functions of the mucosal and muscular layers [42]. Of course, the above-mentioned functional classification of the enteric plexuses is simplified because the previous studies described various types of neuronal cells in each “kind” of enteric plexus. These neurons create short intra-intestinal reflex arcs that regulate the GI tract functioning without the participation of the central nervous system. Until now, sensory intrinsic primary sensory neurons, interneurons, and efferent neurons supplying the muscle, secretory glands, mucosal layer, and intramural blood vessels have been described in all types of the enteric plexuses [35,44]. Nevertheless, the differences in the reaction of the particular types of the ENS plexuses with BPA administration may be the guidance to establish the processes affected by this toxin. For example, in the case of SP, the more visible changes under BPA impact were visible in the OSP and ISP (in comparison to the MP), suggesting that BPA has a greater influence on SP-LI enteric neurons involved in the intestinal secretory activity, immune processes, and/or sensory stimuli conduction than SP-positive enteric cells regulating intestinal muscle contraction.

It should be pointed out that since the impact of BPA on the ENS has not yet been described, the exact mechanisms of the fluctuations in the expression of neuronal factors are still a matter of some conjecture. Moreover, these changes may be connected with various processes including disturbances in the synthesis of neuronal factors on a transcriptional or translational level, as well as inhibition or stimulation of intraneuronal transport.

By analogy to other parts of the nervous system in which the influence of BPA is further described, it can be assumed that the observed changes within the ENS may result from the direct neurotoxicity and neurodegenerative activity of this substance. Such effects of BPA have been investigated in the brain where this toxin causes disturbances in the development of synapses and dendrites, as well as the extension of axons [18,19]. Moreover, BPA alters the monoamine levels in the brain [45] and even a single exposure to this substance may lead to changes in neuroprotein expression [17]. The mechanisms of the above-mentioned BPA activity are probably connected with the capability of this substance to induce changes in gene expression within the nervous tissue by DNA methylation [46].

Interestingly, earlier studies have indicated a significant role of microRNAs (miRNAs) in many biological and pathological processes [47,48] that include, among others, brain development [49,50], as well as reproductive and metabolic functions and dysfunctions [51]. Recent work has shown that BPA has an impact on miRNA expression, which can alter their function in mammals, fish, and cell cultures [51,52]. For example, BPA causes a decrease in the expression of miR-21, which plays an important role in cancer development, such as breast and pancreatic cancer [53,54]. Moreover, it can alter fetal ovarian miRNAs after prenatal BPA treatment in sheep [55]. Furthermore, many specific miRNAs may play an important role in the central nervous system [50]. For instance, miR-134 suppresses the local production of the protein kinase *Limk1* to control spine size [56] and miR-430 recovers brain defects in mutant embryos [57]. Even though it is known that they are important in early neurodevelopment, knowledge about the role of miRNAs in the growth of the nervous system in the prenatal period is scanty and potential mechanisms of BPA action on miRNA expression requires further study [52].

The majority of the neuronal substances investigated in this study are known as neuroprotective factors. Such activity has been described in the case of VIP, which promotes the survivability of neurons after axotomy and toxin exposure to lipopolysaccharide by the stimulation of glial cells [58]. In turn, GAL plays neuroprotective roles in the brain [59] and peripheral nervous system [60], especially during traumatic brain injury, cerebral focal and neurodegenerative diseases [61,62]. These observations together with previous studies on the ENS, where the increase in synthesis of GAL has been described during various diseases [63], strongly suggest the similar neuroprotective functions of this substance in the intestine. The next neuronal factor taking part in neuroprotective processes is SP, which, among others, has been proven to protect neurons from apoptosis and potassium deprivation-induced cell death [64,65]. CART, whose functions in the stomach and intestine are not fully understood, is also known as a strong neuroprotective factor. It protects neuronal cells against focal cerebral ischemia and oxygen–glucose deprivation-induced death, as well as during Parkinson’s disease [66]. There is a strong likelihood that similar processes take place in the ENS, and fluctuations in the expression of neuronal factors noted during the present study are the response to the neurodegenerative influence of BPA.

Contrary to other substances, the percentage of neurons immunoreactive to VAChT decreased after BPA administration. VAChT is one of the markers of neurons synthesizing acetylcholine—the main neurotransmitter within the ENS, which has primarily excitatory functions. Acetylcholine stimulates intestinal motility and secretory activity of the GI tract [35]. It is also known that this substance may show neuroprotective activities in various parts of the nervous system [67,68,69] and may act as an anti-inflammatory factor in the intestine [70,71]. The decrease in the levels of cholinergic neurons observed during the present study suggests a neurotoxic and/or neurodegenerative impact of BPA on the enteric neurons because the results are similar to previous studies on the central nervous system where a decrease in the number of cholinergic neurons during neurodegenerative diseases was noted [72]. It should be underlined that the previous studies on the ENS are inconclusive and suggest multidirectional roles of acetylcholine during pathological processes. It has been demonstrated that the character of changes in the percentage of enteric neurons immunoreactive to VAChT clearly depends not only on the type of inflammatory processes but also on the “kind” of enteric plexus [73,74].

On the other hand, the fluctuations observed in the ENS in this study may be due to the pro-inflammatory impact of BPA and its capacity to modifications within the immunological system. This supposition is supported by a BPA-induced increase in the pro-inflammatory cytokine levels, which have been reported both in the present investigation and in previous studies [75]. It is also known that BPA, as an endocrine disruptor affecting the estrogen receptors, causes a decrease in the number of T and B cells [76]. Furthermore, the close cooperation between the ENS and the intestinal immunological system, as well as the impact of neuronal active substances on immune cells, has been described in previous studies [77,78,79,80]. Some of the neuronal factors studied show pro-inflammatory activity. Namely, SP may affect the NK1-type receptors localized on the surface of lymphocytes and macrophages and, in consequence, promote the secretion of pro-inflammatory factors, including TNF-α [77]. In turn, VIP and GAL appear to be anti-inflammatory factors in the intestine. VIP modulates the balance between anti- and pro-inflammatory cytokines [78] and galanin affects NK cells and increases the levels of IFN-γ and IL-12/23 [79]. Moreover, it may also decrease the levels of TNF- α and IL-1β [80,81]. It should be underlined that the impact of BPA on cytokine expression is not clear. The fluctuations in the neurochemical characteristics of intestinal neuronal cells observed in this investigation may be connected with BPA-induced inflammatory processes and immunological mechanisms. This is supported by the observed increased levels of pro-inflammatory cytokines (including TNF-α, IL-1α, and IL-1β) during the present study after BPA administration and the histopathological changes that are noted in the wall of the ileum where (especially under high doses of BPA) eosinophil and neutrophil infiltration occurred. Interestingly, an increase in neo-immunoreactivity to neuronal factors (which indicates both pro- and anti-inflammatory activity) was observed under the impact of BPA during the present study, but this is in accordance with previous studies on the ENS where such a situation was noted during various types of inflammatory processes [27,28,39,41].

The changes in neurochemical substance levels in the ENS noted in this investigation could also be connected with other mechanisms. One of them is the direct influence of BPA on smooth muscles. Previous studies have shown that BPA demonstrates relaxatory effects on coronary smooth muscle and inhibits the spontaneous contraction of cardiac atrial muscle. The mechanisms of these actions are probably connected with BPA-induced activation of Maxi-K (KCa.1.1) channels in the muscle tissue and the nitric oxide (NO)-dependent guanylyl cyclase (GC) signaling pathway [82,83]. Knowledge of the influence of BPA on the intestinal motility is extremely scanty and limited to one investigation where the relaxatory effects of this toxin on the duodenal contractility were described during ex vivo studies [84]. The mechanisms of this phenomenon remain incompletely elucidated but it is assumed that the mechanisms are connected with nitric oxide-mediated soluble guanylyl cyclase and α-adrenergic signaling pathways [84]. The fluctuations in the neo-immunoreactivity observed during the present study appear to confirm that BPA also has relaxatory effects on porcine ileum. This theory is supported by the increased percentage of neurons that are immunoreactive to VIP and/or CART, as well as a decrease in the number of cholinergic neurons. VIP is one of the most important intestinal inhibitory factors [85] and CART (in spite of incomplete knowledge on the subject) is probably involved in nitric oxide-dependent relaxation in some fragments of the intestine [86]. Contrary to VIP and CART, acetylcholine is the main neuromediator stimulating the contraction of intestinal muscles [35] and a decrease in the number of VAChT-LI neurons occurs during the processes accompanied by the inhibition of intestinal motility. In turn, an increase in the percentage of myenteric neurons immunoreactive to GAL may be connected with the participation of this peptide in the inhibition of the cholinergic pathway via GAL-R1 receptors [87]. Interestingly, the number of myenteric neurons immunoreactive to SP—one of the substances that in light of previous studies causes the contraction of intestinal muscles [88]—has also been observed during the present study. However, these changes may result from a decrease in the intraneuronal transport of SP from cell bodies to nerve endings or may not concern motor neurons but other types of myenteric neurons, including intrinsic primary sensory neurons and/or interneurons. It is even more likely that SP participates in sensory conduction; previous studies have described SP-positive sensory neurons in MP [27,37].

Another cause of the observed changes may be connected with sensory and pain stimuli conduction. Among the substances studied in the present experiment, SP is the most important factor involved in the transmission of sensory stimuli [37,89]. Other neuromediators and/or neuromodulators included in the study also have been described in sensory neurons, although their functions in this part of the nervous system are less relevant and not fully elucidated [27,90,91]. Changes in the levels of neuronal substances, together with histopathological changes noted in the ileal wall (especially abscesses noted after administration of high doses of BPA), could suggest the occurrence of pain. On the other hand, it should be pointed out that the BPA doses administered to the animals in this study were relatively low and should not cause pain signals. Furthermore, during the study, such signals were not observed in the experimental animals. In light of all the facts, the theory that pain is the main cause of the observed changes is unlikely.

The observed changes may also be connected with the influence of BPA on the receptors of the neuronal factors studied but knowledge about this aspect of BPA activity is extremely limited. At present, all that is known is that BPA may downregulate the levels of gene expression of galanin receptor 2 (GAL-R2) and the decrease in the number of receptors may result in the compensatory increase in the synthesis of the substance acting on them [92].

It should be pointed out that the dose of BPA at the level of 0.05 mg/kg body weight/day for a long time was the tolerable daily intake (TDI) established by the provisions of the European Union [93] and, for this reason, was chosen for this study. The tenfold higher dose that was also examined was to determine whether it would cause changes far removed from any changes caused by the low dose. Due to some investigations on the influence of BPA on immune cells, the European Food Safety Authority (EFSA) decided in 2015 to temporarily reduce the tolerable daily intake of BPA from 50 to 4 µg/kg b.w./day [94]. Determination of the definite TDI for BPA depends on further studies on BPA toxicity. The present study may contribute to establishing the final TDI for BPA.

To summarize, the results of this study show that BPA, even in low doses, may affect the ENS of the ileum. The observed changes are probably the first subclinical symptoms of BPA poisoning of the organism and are connected with adaptive and protective processes aimed at maintaining homeostasis in the ileal wall disturbed under the influence of this substance. Admittedly, the exact mechanisms of the observed changes are not clear and learning them requires further study; however, the results of the present study suggest a revision of the views of low doses of BPA.

## 4. Materials and Methods

### 4.1. Experimental Animals and Tissue Collection

This investigation was made up of 15 female pigs of the Piétrain x Duroc breed (at the age of eight weeks, 18–20 kg body weight). The choice of one gender of animals allowed the currently unknown sex-dependent changes in the expression of active substances studied within the ENS to be eliminated. Pigs were kept in standard playpens and all experimental procedures were performed according to the instructions of the local ethics committee responsible for the experimental animals in Olsztyn (Poland) (decision number 17/2013).

Pigs were split into three groups (five animals in each): a C group was maintained in physiological conditions and given empty capsules; an LD group received capsules with BPA at a dose of 0.05 mg/kg b. w./day; and an HD group received capsules with BPA at a dose ten times higher (0.5 mg/kg b. w./day). All groups were given capsules once a day during morning foraging.

After 28 days of BPA consumption, the pigs were premedicated using Stresnil (Janssen, Beerse, Belgium, 75 μL/kg of b. w., given intramuscularly) and subjected to euthanasia by an overdose of sodium thiopental (Thiopental, Sandoz, Kundl-Rakúsko, Austria). Fragments of the ileum (ca. 8 cm long), located 2 cm before the ileocecal valve, were collected. The fragments of the ileum were put into a 10% formalin solution and sent to the Histopathological Laboratory at the Voivodship Children’s Hospital in Olsztyn to evaluate the microscopic changes caused by BPA.

### 4.2. Determination of Neurochemical Characteristics of Enteric Neurons

Ileal fragments (ca. 1 cm long) were fixed in 4% buffered paraformaldehyde (pH 7.4) for one hour, put in phosphate buffer for 72 h, and stored in 18% phosphate-buffered sucrose. After three weeks, tissues were frozen at −22 °C and cut perpendicular to the gut lumen into 14 μm thick sections with a cryostat (HM 525, Microm International, Dreieich, Germany).

Fragments of the ileum were subjected to the typical double-labeling immunofluorescence method described previously by Wojtkiewicz et al. [95]. Slices were incubated (overnight in a humid chamber) with mixtures of antisera received from different mammalian species and directed against protein gene product 9.5 (PGP 9.5, a pan-neuronal marker), substance P (SP), vasoactive intestinal polypeptide (VIP), galanin (GAL), vesicular acetylcholine transporter (VAChT, marker of cholinergic neurons), and cocaine- and amphetamine-regulated transcript peptide (CART). The next day, the tissues were incubated (1 h) with species-specific secondary antibodies linked to various fluorochromes. The specifications of the antibodies are detailed in Table 2. Pre-absorption of the antibodies with appropriate antigens, as well as routine omission and replacement tests, were made up to exclude non-specific labeling.

Ileal fragments were studied using an Olympus BX51 microscope equipped with an appropriate filter set for immunofluorescence. To determine the percentage of neurons showing the presence of the particular neuronal factors, at least 500 PGP-9.5-LI neurons were assessed with regard to the presence of each neuronal factor studied (the number of PGP 9.5 was considered as 100%). To prevent double evaluation of the same neurons, the fragments of the ileum included in the investigation were located at least 200 µm apart from each other. The data were pooled and are presented as the mean ± SEM.

### 4.3. Determination of Interleukin Levels by ELISA 

After resection of the fragment of ileum, Peyer’s patches were collected using the mechanical macroscopic method described previously by Obremski et al. [96], fixed in liquid nitrogen, and then stored at −80 °C until analysis.

Using a homogenizer (Omni-TipsTM Disposable, Omni International, Kennesaw, USA), 1 g of ileal Peyer’s patches were homogenized in 2.5 mL of extraction buffer with the following composition: PBS (137 mM NaCl, 2.7 mM KCl, 8.1 mM Na_2_HPO_4_, 1.5 mM KH_2_PO_4_), 0.5% sodium citrate, 0.05% (POCH, Gliwice, Poland), Tween 20 (Sigma Aldrich, Saint Louis, MO, USA), and protease inhibitors (Ref. 11 697 498 001, Roche, Basel, Switzerland). The homogenate was centrifuged for 60 min at 8600× *g* (Eppendorf 5804 R) and the obtained supernatant was subjected to routine ELISA tests (Porcine IL-1 alpha/IL-1F1 DuoSet ELISA, R&D Systems, Inc., Minneapolis, MN, USA; Porcine IL-1 beta/IL-1F2 DuoSet ELISA, R&D Systems; Porcine TNF-alpha DuoSet ELISA, R&D Systems). Tissue concentrations of cytokines (IL-1α, IL-1β, TNF-α) were determined by ELISA using commercial kits in accordance with the manufacturer’s instructions. The concentration of cytokines was determined by an ELISA kit using a TECAN multifunctional plate reader (TECAN Infinite M200 Plate Reader Tecan, Männedorf, Switzerland).

ELISA polystyrene 96-well microplates (NUNC, Denmark) were coated with a solution of capture antibodies in carbonate buffer (100 µL per well, overnight, at 4 °C). After this step, the plate was washed three times in PBS with 0.05% Tween 20 (tPBS) and blocked in 1% BSA (Sigma Aldrich, Saint Louis, MO, USA) in PBS (90 min at 37 °C). Afterward, the extract samples and standards were added and incubated for 90 min. Next, a solution of detection antibodies in PBS with 1% BSA (Sigma Aldrich, USA) was added and incubated for 90 min. After this time, a solution of streptavidin conjugated with HRP enzyme was added. The next step was the incubation with substrate (OPD or TMB, Sigma Aldrich, USA) for 30 min. The enzymatic reaction was stopped by the addition of 2.5 M HCl (POCH, Gliwice, Poland). The plate was then measured in a plate reader at 492 nm or 450 nm (depending on the substrate OPD or TMB).

### 4.4. Statistical Analysis

The statistical analysis was performed with one-way analysis of variance (ANOVA) with Bonferroni’s Multiple Comparison post hoc test using Statistica 12 software (StatSoft Inc., Tulsa, OK, USA). Differences were considered significant at *p* < 0.05 (*) and as highly significant at *p* ≤ 0.01 (**) and *p* ≤ 0.001 (***).

## Figures and Tables

**Table 1:** The percentage (mean ± SEM) of neurons showing the presence of the chosen neuronal active substances.

Part of ENS	Animal Group	Active Neuronal Substances Studied During the Experiment				
		VAChT	VIP	SP	GAL	CART
**Myenteric plexus**	C group	18.35 ± 0.21	15.05 ± 0.17	11.01 ± 0.16	7.91 ± 0.04	7.58 ± 0.39
	LD group	17.54 ± 0.24 *	15.21 ± 0.35	11.03 ± 0.47	8.06 ± 0.32	10.70 ± 0.40 ***
	HD group	13,67 ± 0.18 ***	17.51 ± 0.04 ***	15.15 ± 0.28 ***	13.40 ± 0.39 ***	17.09 ± 0.52 ***
**Outer submucous plexus**	C group	19.93 ± 0.10	10.57 ± 0.39	9.76 ± 0.12	9.60 ± 0.41	4.47 ± 0.11
	LD group	18.24 ± 0.23 ***	12.61 ± 0.11 ***	10.39 ± 0.32	12.92 ± 0.42 ***	5.29 ± 0.36
	HD group	14.70 ± 0.12 ***	16.00 ± 0.08 ***	17.73 ± 0.33 ***	18.08 ± 0.35 ***	9.65 ± 0.20 ***
**Inner submucous plexus**	C group	26.42 ± 0.38	7.64 ± 0.17	9.32 ± 0.37	14.14 ± 0.13	3.80 ± 0.16
	LD group	25.21 ± 0.23 *	10.85 ± 0.34 ***	10.32 ± 0.31	18.17 ± 0.08 ***	5.30 ± 0.24 ***
	HD group	20.70 ± 0.11 ***	17.80 ± 0.28 ***	15.83 ± 0.42 ***	22.72 ± 0.30 ***	8.02 ± 0.04 ***

Vesicular acetylcholine transporter (VAChT), vasoactive intestinal polypeptide (VIP), substance P (SP), galanin (GAL), or cocaine- and amphetamine-regulated transcript peptide (CART) in relation to protein gene product 9.5 (PGP 9.5)-positive neurons stained for PGP 9.5 in the control (C) group and after administration of low (LD group) and high (HD) doses of bisphenol A (BPA). Statistically significant (*p* ≤ 0.05) and highly statistically significant (*p* ≤ 0.001) differences between the C group and LD group, as well as the C group and HD group, are marked with * and ***.

**Table 2:** Reagents used in immunofluorescence method.

**Primary Antibody**				
**Antisera**	**Code**	**Host Species**	**Dilution**	**Supplier**
PGP9.5	7863-2004	Mouse	1:1000	Bio-Rad, Hercules, CA, USA
CART	H-003-61	Rabbit	1:16,000	Phoenix Pharmaceuticals, Inc., Burlingame, CA, USA
GAL	AB2233	Guinea Pig	1:2000	EMD Millipore, Burlington, MA, USA
SP	8450-0505	Rat	1:1000	Bio-Rad
VAChT	H-V007	Rabbit	1:2000	Phoenix Pharmaceuticals
VIP	9535-0204	Mouse	1:2000	Bio-Rad
**Secondary antibodies**				
**Reagent**			**Dilution**	**Supplier**
AF 488 donkey anti-mouse IgG (H + L)			1:1000	Thermo Fisher Scientific, Waltham, MA, USA
AF 546 goat anti-guinea pig IgG (H + L)			1:1000	Thermo Fisher Scientific
AF 546 goat anti-rabbit IgG (H + L)			1:1000	Thermo Fisher Scientific
AF 546 goat anti-rat IgG (H + L)			1:1000	Thermo Fisher Scientific

PGP9.5 (pan-neuronal marker); CART, cocaine- and amphetamine-regulated transcript; GAL, galanin; SP, substance P; VAChT, vesicular acetylcholine transporter; VIP, vasoactive intestinal peptide; AF, Alexa Fluor.
